# *Hsa-miR-34b/c* rs4938723 T>C and *hsa-miR-423* rs6505162 C>A Polymorphisms Are Associated with the Risk of Esophageal Cancer in a Chinese Population

**DOI:** 10.1371/journal.pone.0080570

**Published:** 2013-11-18

**Authors:** Jun Yin, Xu Wang, Liang Zheng, Yijun Shi, Liming Wang, Aizhong Shao, Weifeng Tang, Guowen Ding, Chao Liu, Ruiping Liu, Suocheng Chen, Haiyong Gu

**Affiliations:** 1 Department of Cardiothoracic Surgery, Affiliated People’s Hospital of Jiangsu University, Zhenjiang, China; 2 Department of Cardiothoracic Surgery, The First People’s Hospital of Changzhou and The Third Affiliated Hospital of Suzhou University, Changzhou, China; 3 Cancer institute, Department of Chemotherapy, Affiliated People’s Hospital of Jiangsu University, Zhenjiang, Jiangsu, China; 4 Department of Orthopedics, Affiliated Hospital of Nanjing Medical University, Changzhou Second People’s Hospital, Changzhou, China; The University of Hong Kong, China

## Abstract

Esophageal cancer is the eighth most common cancer and sixth leading cause of cancer associated death worldwide. Besides environmental risk factors, genetic factors might play an important role in the esophageal cancer carcinogenesis. We conducted a hospital based case–control study to evaluate the genetic susceptibility of functional single nucleotide polymorphisms (SNPs) in the microRNAs on the development of esophageal cancer. A total of 629 esophageal squamous cell carcinoma (ESCC) cases and 686 controls were recruited for this study. The *hsa-miR-34b/c* rs4938723 T>C, *pri-miR-124-1* rs531564 C>G, *pre-miR-125a* rs12975333 G>T and *hsa-miR-423* rs6505162 C>A genotypes were determined using Ligation Detection Reaction (LDR) method. Our results demonstrated that *hsa-miR-34b/c* rs4938723 CC genotype had a decreased risk of ESCC. The association was evident among patients who never drinking. *Hsa-miR-423* rs6505162 C>A might associated with a significantly increased risk of ESCC in patients who smoking. These findings indicated that functional polymorphisms *hsa-miR-34b/c* rs4938723 T>C and *hsa-miR-423* rs6505162 C>A might alter individual susceptibility to ESCC. However, our results were obtained with a limited sample size. Future larger studies with other ethnic populations are required to confirm current findings.

## Introduction

MicroRNAs (miRNAs) are tiny noncoding RNAs that act as posttranscriptional gene regulatory elements [1]. Specifically, miRNAs act by binding to the 3’-untranslated region of target genes and to consequently down-regulate their expression [2]. MiRNAs are important players in carcinogenesis [3].

Genetic factors, such as single nucleotide polymorphisms (SNPs), may contribute to carcinogenesis [4]. The SNPs in the genomic miRNA sequences could influence miRNA-dependent regulation, affect the final level and function of miRNAs and alter consequently tumor susceptibility [5].

Members of the miR-34 family are direct p53 targets, and their expression is directly induced by p53 in response to DNA damage or oncogenic stress [6]. In previous studies in colorectal cancer [7], oral cancer [8] and malignant melanoma [9], down-regulation of mir-34b/c by methylation was found. *Hsa-miR-34b/c* rs4938723 polymorphism is located within the CpG island of pri-miR-34b/c, and might be the predicted binding site for GATA-X transcription factors [10]. *Hsa-miR-34b/c* rs4938723 T>C polymorphism was associated with the risk of nasopharyngeal carcinoma [11], hepatocellular carcinoma [12], colorectal cancer [13] and breast cancer survival [14].

Besides *hsa-miR-34b/c* rs4938723 T>C, *pri-miR-124-1* rs531564 C>G, *pre-miR-125a* rs12975333 G>T and *hsa-miR-423* rs6505162 C>A were also associated with the risk of different types of cancers. The rs531564 C>G SNP in *pri-miR-124-1* was associated with an increased risk of bladder cancer [15] and esophageal cancer in males [16]. The *pre-miR-125a* rs12975333 G>T polymorphism was a founder mutation specific to the Antwerp area and associated with high risk for breast cancer [17]. *Hsa-miR-423* rs6505162 C>A polymorphism was associated with reduced breast cancer risk [18]. *Hsa-miR-423* rs6505162 C>A polymorphism was also significantly associated with both the overall survival and the recurrence-free survival of colorectal cancer [19].

We previously investigated *miR-196a2* rs11614913 T>C, *miR-146a* rs2910164 C>G, *miR-499* rs3746444 T>C, *miR-26a-1* rs7372209 C>T and *miR-27a* rs895819 T>C SNPs and esophageal squamous cell carcinoma (ESCC) risk in 380 cancer cases and 380 controls [20]. We found *miR-196a2* rs11614913 T>C might contribute to decreased ESCC risk among women patients and patients who never smoking or drinking [20]. Now, the objective of this investigation was to evaluate the association between *hsa-miR-34b/c* rs4938723 T>C, *pri-miR-124-1* rs531564 C>G, *pre-miR-125a* rs12975333 G>T and *hsa-miR-423* rs6505162 C>A genotypes and ESCC risk. We performed genotyping analyses for the four *miRNA* SNPs with 629 ESCC cases and 686 controls in a Chinese population.

## Materials and Methods

### Ethical approval of the study protocol

This hospital-based case-control study was approved by the Review Board of Jiangsu University (Zhenjiang, China). We have complied with the World Medical Association Declaration of Helsinki regarding ethical conduct of research involving human subjects and/or animals. All subjects provided written informed consent to be included in the study.

### Patients and Controls

629 subjects with esophageal cancer were consecutively recruited from the Affiliated People’s Hospital of Jiangsu University and Affiliated Hospital of Jiangsu University (Zhenjiang, China) between October 2008 and December 2010. Diagnosis was done by biopsy and all cases of esophageal cancer were ESCC. The exclusion criteria were patients who previously had: cancer; any metastasized cancer; radiotherapy or chemotherapy. The 686 controls were patients without cancer frequency-matched to the cases with regard to age (±5 years) and sex recruited from the two hospitals mentioned above during the same time period. Most of the controls were admitted to the hospitals for the treatment of trauma.

Each subject was personally questioned by trained interviewers using a pre-tested questionnaire to obtain information on demographic data (e.g., age, sex) and related risk factors (including tobacco smoking and alcohol consumption). After the interview, 2-mL samples of venous blood were collected from each subject. Individuals who smoked one cigarette per day for >1 year were defined as “smokers”. Subjects who consumed ≥3 alcoholic drinks a week for >6 months were considered to be “alcohol drinkers”.

### Isolation of DNA and genotyping by Ligation Detection Reaction

Blood samples were collected from patients using Vacutainers and transferred to tubes lined with ethylenediamine tetra-acetic acid (EDTA). Genomic DNA was isolated from whole blood with the QIAamp DNA Blood Mini Kit (Qiagen, Berlin, Germany) [21]. Sample DNA were amplified by PCR according to the manufacturer’s recommendations. The samples were genotyped using the Ligation Detection Reaction (LDR) method with technical support from the Shanghai Biowing Applied Biotechnology Company as previously described [22]. For quality control, repeated analyses were done for 160 (12.17%) randomly selected samples with high DNA quality.

### Statistical Analyses

Differences in the distributions of demographic characteristics, selected variables, and genotypes of the *hsa-miR-34b/c* rs4938723 T>C, *pri-miR-124-1* rs531564 C>G, *pre-miR-125a* rs12975333 G>T and *hsa-miR-423* rs6505162 C>A variants between the cases and controls were evaluated using student t test and the *χ* ^2^ test. The associations between the four SNPs and risk of ESCC were estimated by computing the ORs and their 95% CIs using logistic regression analyses for crude ORs and adjusted ORs when adjusting for age, sex, smoking and drinking status. The Hardy-Weinberg equilibrium (HWE) was tested by a goodness-of-fit *χ* ^2^ test to compare the observed genotype frequencies to the expected ones among the control subjects. All statistical analyses were performed with SAS 9.1.3 (SAS Institute, Cary, NC, USA).

## Results

### Characteristics of the study population

Characteristics of cases and controls included in the study are summarized in Table 1. The cases and controls appeared to be adequately matched on age and sex as suggested by the *χ* ^2^ tests (*p* = 0.541 and *p* = 0.185, respectively). As shown in Table 1, significant difference was detected on smoking status between the cases and the controls (*p* < 0.001), and drinking rate was higher in ESCC patients than in control subjects (*p* < 0.001). The primary information for four genotyped SNPs was in Table 2. The genotyping success rate is ranging from 95.13% to 96.81% in all 1315 samples. The concordance rates of repeated analyses were 100% for all four SNPs. Minor allele frequency (MAF) in our controls was similar to MAF for Chinese in database for all four SNPs (Table 2). The observed genotype frequencies for *hsa-miR-34b/c* rs4938723 T>C, *pri-miR-124-1* rs531564 C>G and *hsa-miR-423* rs6505162 C>A polymorphisms in the controls were in HWE (*p* = 0.675, *p* = 0.400 and *p* = 0.299) except *pre-miR-125a* rs12975333 G>T (not available) (Table 2).

**Table 1:** Distribution of selected demographic variables and risk factors in ESCC cases and controls.

Variable	Cases (n = 629) n %		Controls (n = 686) n %		*p* a
**Age (years)** mean ± SD	62.85 (±8.13)		62.58 (±7.89)		0.541
**Age (years)**					0.155
< 63	310	49.28	365	53.21	
≥ 63	319	50.72	321	46.79	
**Sex**					0.185
Male	444	70.59	461	67.20	
Female	185	29.41	225	32.80	
**Tobacco use**					**<0.001**
Never	355	56.44	499	72.74	
Ever	274	43.56	187	27.26	
**Alcohol use**					**<0.001**
Never	428	68.04	526	76.68	
Ever	201	31.96	160	23.32	

a Two-sided *χ* ^2^ test and student t test; Bold values are statistically significant (*p* <0.05).

**Table 2:** Primary information for *hsa-miR-34b/c* rs4938723 T>C, *pri-miR-124-1* rs531564 C>G, *pre-miR-125a* rs12975333 G>T and *hsa-miR-423* rs6505162 C>A polymorphisms.

Genotyped SNPs	*hsa-miR-34b/c* rs4938723 T>C	*pri-miR-124-1* rs531564 C>G	*pre-miR-125a* rs12975333 G>T	*hsa-miR-423* rs6505162 C>A
Chromosome	11	8	19	17
Gene Official Symbol	MIR34B/C	MIR124-1	MIR125A	MIR423
Function	ncRNA	ncRNA	ncRNA	ncRNA
Chr Pos (Genome Build 36.3)	110887775	9798109	56888340	25468309
Regulome DB Score^a^	5	5	5	1f
TFBS^b^	Y	Y	Y	Y
Splicing (ESE or ESS)	—	—	—	Y
MAF^c^ for Chinese in database	0.400	0.178	Unknown	0.200
MAF in our controls (n = 686)	0.324	0.157	0.000	0.188
*p* value for HWE^d^test in our controls	0.675	0.400	—	0.299
Genotyping method^e^	LDR	LDR	LDR	LDR
% Genotyping value	96.81%	96.43%	96.43%	95.13%

a http://www.regulomedb.org/;

b TFBS: Transcription Factor Binding Site (http://snpinfo.niehs.nih.gov/snpinfo/snpfunc.htm);

c MAF: minor allele frequency;

d HWE: Hardy–Weinberg equilibrium;

e LDR: Ligation Detection Reaction.

### Associations between hsa-miR-34b/c rs4938723 T>C, pri-miR-124-1 rs531564 C>G, pre-miR-125a rs12975333 G>T and hsa-miR-423 rs6505162 C>A polymorphisms and risk of ESCC

The genotype distributions of *hsa-miR-34b/c* rs4938723 T>C, *pri-miR-124-1* rs531564 C>G, *pre-miR-125a* rs12975333 G>T and *hsa-miR-423* rs6505162 C>A in the cases and the controls are shown in Table 3. In the single locus analyses, the genotype frequencies of *hsa-miR-34b/c* rs4938723 T>C were 46.2% (TT), 46.3% (TC), and 7.5% (CC) in the case patients and 46.1% (TT), 43.1% (TC), and 10.8% (CC) in the control subjects, and the difference was not statistically significant (*p* = 0.101). In the recessive model, when the *hsa-miR-34b/c* rs4938723 TT/TC genotypes were used as the reference group, the CC homozygote genotype was associated with a statistically significantly decreased risk for ESCC (CC vs. TT/TC: adjusted OR = 0.65, 95% CI = 0.44–0.97, *p* = 0.036). When the *hsa-miR-34b/c* rs4938723 TT homozygote genotype was used as the reference group, the TC genotype was not associated with the risk for ESCC (TC vs. TT: adjusted OR = 1.11, 95% CI = 0.88–1.40, *p* = 0.397); the CC genotype was not associated with the risk for ESCC (CC vs. TT: adjusted OR = 0.69, 95% CI = 0.45–1.04, *p* = 0.076). In the dominant model, the *hsa-miR-34b/c* rs4938723 TC/CC variants were not associated with the risk for ESCC, compared with the *hsa-miR-34b/c* rs4938723 TT genotype (adjusted OR = 1.02, 95% CI = 0.82–1.28, *p* = 0.853) (Table 3).

**Table 3:** Logistic regression analyses of associations between *hsa-miR-34b/c* rs4938723 T>C, *pri-miR-124-1* rs531564 C>G, *pre-miR-125a* rs12975333 G>T and *hsa-miR-423* rs6505162 C>A polymorphisms and risk of ESCC.

Genotype	Cases (n = 629) n %				Controls(n = 686) n %		Crude OR(95%CI)	*p*	Adjusted OR a(95%CI)	*p*
*hsa-miR-34b/c*rs4938723 T>C										
TT	277	46.2		310		46.1	1.00		1.00	
TC	278	46.3		290		43.1	1.07 (0.85–1.35)	0.551	1.11 (0.88–1.40)	0.397
CC	45	7.5		73		10.8	0.69 (0.46–1.04)	0.073	0.69 (0.45–1.04)	0.076
CC vs. TC vs. TT										0.101
TC+CC	323	53.8		363		53.9	1.00 (0.80–1.24)	0.970	1.02 (0.82–1.28)	0.853
TT+TC	555	92.5		600		89.2	1.00		1.00	
CC	45	7.5		73		10.8	**0.67 (0.45**–**0.98)**	**0.041**	**0.65 (0.44**–**0.97)**	**0.036**
T allele	832	69.3		910		67.6	1.00			
C allele	368	30.7		436		32.4	0.92 (0.78–1.09)	0.350		
*pri-miR-124-1*rs531564 C>G										
CC	454	74.3		470		71.5	1.00		1.00	
CG	146	23.9		168		25.6	0.90 (0.70–1.16)	0.726	0.96 (0.74–1.25)	0.768
GG	11	1.8		19		2.9	0.60 (0.28–1.27)	0.183	0.63 (0.29–1.36)	0.237
GG vs. CG vs. CC										0.319
CG+GG	157	25.7		187		28.5	0.87 (0.68–1.11)	0.269	0.93 (0.72–1.19)	0.559
CC+CG	600	98.2		638		97.1	1.00		1.00	
GG	11	1.8		19		2.9	0.62 (0.29–1.30)	0.205	0.64 (0.30–1.37)	0.245
C allele	1054	86.3		1108		84.3	1.00			
G allele	168	13.7		206		15.7	0.86 (0.69–1.07)	0.171		
*pre-miR-125a*rs12975333 G>T										
GG	611	100.0		657		100.0	1.00		1.00	
GT	0	0.0		0		0.0	—	—	—	—
TT	0	0.0		0		0.0	—	—	—	—
*hsa-miR-423*rs6505162 C>A										
CC	374	62.3		425		65.3	1.00		1.00	
CA	197	32.8		207		31.8	1.08 (0.85–1.37)	0.522	1.09 (0.86–1.40)	0.476
AA	29	4.8		19		2.9	1.73 (0.96–3.14)	0.070	1.70 (0.92–3.12)	0.089
AA vs. CA vs. CC										0.173
CA+AA	226	37.7		226		34.7	1.14 (0.90–1.43)	0.278	1.14 (0.90–1.45)	0.264
CC+CA	571	95.2		632		97.1	1.00		1.00	
AA	29	4.8		19		2.9	1.69 (0.94–3.05)	0.081	1.65 (0.90–3.01)	0.106
C allele	945	78.8		1057		81.2	1.00			
A allele	255	21.3		245		18.8	1.16 (0.96–1.42)	0.128		

a Adjusted for age, sex, smoking status and alcohol consumption.

No association was observed between *pri-miR-124-1* rs531564 C>G and *hsa-miR-423* rs6505162 C>A polymorphisms and the risk of ESCC (Table 3). For *pre-miR-125a* rs12975333 G>T, all genotypes are GG homozygotes (Table 3).

### Stratification analyses of hsa-miR-34b/c rs4938723 T>C, pri-miR-124-1 rs531564 C>G and hsa-miR-423 rs6505162 C>A and risk of ESCC

To evaluate the effects of *hsa-miR-34b/c* rs4938723 T>C, *pri-miR-124-1* rs531564 C>G and *hsa-miR-423* rs6505162 C>A genotypes on ESCC risk according to different age, sex, smoking and alcohol drinking status; we performed the stratification analyses (Table 4). A significantly decreased risk of ESCC associated with the *hsa-miR-34b/c* rs4938723 T>C polymorphism was evident among patients who never drinking (CC vs. TT/TC: adjusted OR = 0.57, 95% CI = 0.34–0.94, *p* = 0.029) (Table 4). In patients who smoking, *hsa-miR-423* rs6505162 C>A might associated with a significantly increased risk of ESCC (AA vs. CC/CA: adjusted OR = 4.94, 95% CI = 1.42–17.21, *p* = 0.012) (Table 4).

**Table 4:** Stratified analyses between *hsa-miR-34b/c* rs4938723 T>C, *pri-miR-124-1* rs531564 C>G and *hsa-miR-423* rs6505162 C>A polymorphism and ESCC risk by sex, age, smoking status and alcohol consumption.

Variable	*hsa-miR-34b/c* rs4938723 T>C (case/control)		Adjusted OR (95% CI) a		*pri-miR-124-1* rs531564 C>G (case/control)		Adjusted OR (95% CI)		*hsa-miR-423* rs6505162 C>A (case/control)		Adjusted OR (95% CI)	
	TT+TC	CC	TT+TC	CC	CC+CG	GG	CC+CG	GG	CC+CA	AA	CC+CA	AA
Sex												
Male	396/402	32/47	1.00	0.68(0.42–1.10)	420/430	9/14	1.00	0.69(0.29–1.63)	402/419	24/16	1.00	1.54(0.79–2.98)
Female	159/198	13/26	1.00	0.60(0.30–1.21)	180/208	2/5	1.00	0.51(0.10–2.67)	169/213	5/3	1.00	2.26(0.53–9.66)
Age												
<63	274/319	20/37	1.00	0.64(0.35–1.15)	297/333	4/14	1.00	0.35(0.11–1.11)	278/333	16/12	1.00	1.75(0.79–3.86)
≥63	281/281	25/36	1.00	0.68(0.39–1.16)	303/305	7/5	1.00	1.33(0.41–4.31)	293/299	13/7	1.00	1.91(0.74–4.94)
Smoking status												
Never	309/441	24/51	1.00	0.62(0.37–1.04)	341/460	6/14	1.00	0.68(0.26–1.79)	327/456	10/16	1.00	0.96(0.42–2.20)
Ever	246/159	21/22	1.00	0.69(0.36–1.30)	259/178	5/5	1.00	0.74(0.21–2.64)	244/176	19/3	1.00	**4.94(1.42**–**17.21)**
Alcohol consumption												
Never	379/465	25/54	1.00	**0.57(0.34–0.94)**	408/486	8/14	1.00	0.77(0.31–1.91)	391/484	16/15	1.00	1.41(0.66–2.99)
Ever	176/135	20/19	1.00	0.79(0.39–1.57)	192/152	3/5	1.00	0.50(0.12–2.17)	180/148	13/4	1.00	3.00(0.94–9.57)

a Adjusted for age, sex, smoking status and alcohol consumption (besides stratified factors accordingly) in a logistic regression model; Bold values are statistically significant (*p* <0.05).

## Discussion

In this hospital-based case-control study of ESCC, we investigated the associations of *hsa-miR-34b/c* rs4938723 T>C, *pri-miR-124-1* rs531564 C>G, *pre-miR-125a* rs12975333 G>T and *hsa-miR-423* rs6505162 C>A with risk of ESCC in a high risk Chinese population. Our multivariable logistic analysis revealed that *hsa-miR-34b/c* rs4938723 CC genotype had a decreased risk of ESCC. The association was evident among patients who never drinking. *Hsa-miR-423* rs6505162 C>A might associated with a significantly increased risk of ESCC in patients who smoking.

It is well known that members of the miR-34 family expression is directly induced by p53 in response to DNA damage or oncogenic stress [6]. A rat model experiment indicated that the alteration of miR-34b/c under an inflammatory microenvironment can be influenced by p53 [23]. By DNA methylation of its own promoter, miR-34 family has been silenced in numerous cancers [24]. By triggering Wnt signaling cascades, the loss of miR-34 impairs p53-mediated cell death. Overexpression of miR-34 can induce apoptosis [25]–[27]. The role of miR-34a in the suppression of tumor growth has also been detected in vivo [28]. In a previous study, down-regulation of mir-34b/c by methylation was found in colorectal cancer [7], oral cancer [8] and malignant melanoma [9]. The *hsa-miR-34b/c* rs4938723 T>C SNP is located within the CpG island of pri-miR-34b/c and may affect a predicted GATA-X transcription factor binding [10]. *Hsa-miR-34b/c* rs4938723 T>C polymorphism was associated with the risk of nasopharyngeal carcinoma [11], hepatocellular carcinoma [12], colorectal cancer [13] and breast cancer survival [14]. *Hsa-miR-423* rs6505162 C>A polymorphism was associated with reduced breast cancer risk [18] and both the overall survival and the recurrence-free survival of colorectal cancer [19]. In our study, we found *hsa-miR-34b/c* rs4938723 CC genotype had a decreased risk of ESCC among patients who never drinking, *hsa-miR-423* rs6505162 C>A might associated with a significantly increased risk of ESCC in patients who smoking, indicating gene-environment interaction.

The frequencies of genetic polymorphisms often vary between ethnic groups. In the present Chinese study, the allele frequency of *hsa-miR-34b/c* rs4938723 C was 0.324 among 686 control subjects, which is slightly higher than that of Japanese population (0.261) and similar to that of European population (0.310) and Sub-Saharan African population (0.305). The allele frequency of *hsa-miR-423* rs6505162 A was 0.188 among 686 control subjects, which is in accordance with that of Chinese population (0.200) and Japanese population (0.178). But the allele frequency is significantly lower than that of European population (0.575) and Sub-Saharan African population (0.783) (http://www.ncbi.nlm.nih.gov/SNP, http://hapmap.ncbi.nlm.nih.gov/).

Using Power and Sample Size Calculation (PS, version 3.0, 2009, http://biostat.mc.vanderbilt.edu/twiki/bin/view/Main/PowerSampleSize), considering *hsa-miR-34b/c* rs4938723 T>C mutant alleles in the control group, OR, ESCC samples and control samples, the power of our analysis (α = 0.05) was 0.929 in 600 ESCC cases and 673 controls with adjusted OR 0.65. The power of our analysis (α = 0.05) was 0.962 in 404 ESCC cases and 519 controls with adjusted OR 0.57 in non- drinking subgroup. For *hsa-miR-423* rs6505162 C>A, the power of our analysis (α = 0.05) was 1.000 in 263 ESCC cases and 179 controls with adjusted OR 4.94 in smoking subgroup.

In this case-control study, several limitations need to be addressed. Firstly, the patients and controls were enrolled from hospitals and may not represent the general population. Secondly, statistical power of our study was limited the especially in stratification analyses, it is better that the control group being larger than the case group; therefore it is possible to have a more statistical power. Thirdly, detailed information on cancer metastasis and survival were not recruited till now, which restricted further analyses on the role of the four polymorphisms in ESCC progression and prognosis. Finally, the information about viral infections and immune parameters was not available, which restricted the power of our analyses.

In conclusion, our study provides evidence that functional polymorphisms *hsa-miR-34b/c* rs4938723 T>C and *hsa-miR-423* rs6505162 C>A might alter individual susceptibility to ESCC. Future larger studies with other ethnic populations and functional analysis are required to confirm current findings.
